# Does antiretroviral therapy change partnership dynamics and HIV risk behaviours among HIV-infected adults

**DOI:** 10.1097/QAD.0000000000001502

**Published:** 2017-06-02

**Authors:** Nuala McGrath, Erofili Grapsa

**Affiliations:** a University of Southampton, Southampton, UK; b Africa Health Research Institute, KwaZulu-Natal; c Institute of Social And Economic Research, Rhodes University, Eastern Cape, South Africa.

**Keywords:** Africa, antiretroviral therapy, HIV, partnership dynamics, sexual behaviour

## Abstract

**Objective:**

We explore the impact of antiretroviral therapy (ART) on partnership acquisition and dissolution rates and changes in sexual behaviours among HIV-infected adults.

**Design:**

Using detailed longitudinal data from a prospective cohort of HIV-infected adults with CD4^+^ cell count below 200 cells/μl (ART-eligible) or CD4^+^ cell count above 500 cells/μl (pre-ART) conducted in rural KwaZulu-Natal, South Africa, from 2009 to 2012.

**Methods:**

Partnership acquisition and dissolution are explored through survival analysis methods, whereas generalized linear models were fitted for the sexual behaviour outcomes with interaction terms to allow the association with ART to vary over time. Throughout, the primary comparison of interest for each outcome is differences between the two ART groups.

**Results:**

ART is not associated with partner acquisition or relationship dissolution. During follow-up, the two ART groups do not differ in the odds of being sexually active nor the number of sex acts, whereas the odds of unprotected sex are significantly lower for partnerships of ART-eligible participants (adjusted odds ratio 0.26, 95% confidence interval 0.15, 0.43). Relationship-level characteristics including cohabitation status and wanting more children with that partner are associated with higher odds and increased frequency of sexual activity, and increased odds of unprotected sex, whereas living with partner, higher relationship quality and longer relationship duration are associated with lower risk of partnership dissolution.

**Conclusion:**

Being on ART was not associated with increased sexual risk behaviours, a reassuring finding given the WHO recommends ART initiation upon HIV diagnosis. The importance of relationship-level characteristics provides evidence that HIV care services should offer routine support for HIV disclosure and sexual risk reduction, and promotion of couples-testing and positive couple relationships.

## Introduction

Modelling studies incorporating sexual behaviour change among HIV-infected individuals due to antiretroviral therapy (ART) separately from sexual behaviour change among HIV-uninfected individuals due to the availability of ART predict that even small increases in partner acquisition and partner dissolution rates will reduce the overall impact of ART rollout on HIV incidence at the population level [1,2]. In sub-Saharan Africa, the majority of men and women of reproductive age are in heterosexual monogamous sexual partnerships [3]. Studies examining the risk of partnership dissolution associated with HIV have consistently reported that serodiscordant relationships in which the woman was HIV-positive were the most likely to dissolve [4–6]. Higher dissolution rates are likely to lead to increased acquisition of new partnerships over time. For an HIV-infected adult, forming new partnerships requires repeated HIV disclosure and condom use negotiation to prevent onward HIV transmission. In Africa, few sources of longitudinal data are available with which to estimate partnership acquisition and dissolution rates among HIV-infected individuals, and the impact of ART on these rates.

With respect to the association between ART and subsequent sexual behaviour, a review by Venkatesh *et al.*[7] in 2011 found that only one study out of 17 in African populations reported a finding of higher risk sexual behaviours, specifically increased unprotected sex, among HIV-infected individuals after ART initiation. However, other than cross-sectional reports of type of partnership, partner's HIV status and multiple partnerships, limited partnership characteristics were available for risk factor analysis [8–11]. Relationship dynamics play a role in the acceptability of condoms within partnerships [12], and HIV disclosure [13]. Among HIV serodiscordant couples, desire for future children together, being co-parents of living children and couples without an income where the male was the individual with HIV are associated with lower risk of partnership dissolution [6,14]. Venkatesh *et al.* propose a conceptual model for the way in which partnership (dyadic) factors such as non-disclosure of HIV status and fertility desires are associated with increased sexual risk behaviours, whereas other partnership factors such as condom use within the partnership are associated with decreased sexual risk behaviours in the context of ART availability [7].

We use detailed longitudinal data from a prospective cohort conducted in rural KwaZulu-Natal, South Africa, between 2009 and 2012, to investigate the impact of ART on partnership acquisition and dissolution rates among HIV-infected individuals. We also examine the changes in sexual behaviours, specifically sexual activity, unprotected sex and levels of sexual activity, associated with taking ART.

## Methods

The cohort study has been described elsewhere [15,16]. Men and women attending one of three primary healthcare clinics within the HIV Treatment and Care Programme in the Hlabisa sub-district of Umkhanyakude in northern KwaZulu Natal, South Africa [17], with CD4^+^ cell count less than 200 cells/μl (‘ART-eligible’ at enrolment) or CD4^+^ cell count above 500 cells/μl (ART-ineligible at enrolment, referred to as ‘pre-ART’) were eligible for the study between January 2009 and March 2011 if resident within the Africa Centre Demographic Surveillance Area, and not currently pregnant (women). A questionnaire was administered at enrolment and 6-monthly through 36 months, or October 2012. Demographic and social variables, and also details regarding up to three sexual partnerships in the past 6 months, sexual activity and condom use data were collected at each study visit. Participants who reported an ongoing partnership were asked additional questions about the quality of those relationships and their fertility intentions with their current main partner. Scales from the literature were adapted to measure gender norms [18], HIV stigma [19] and relationship quality [20]. Further details are given in the study by Fladseth *et al.*[21]. This study addresses one of the specific objectives of the cohort study, to compare sexual behaviour and partner change over a 3-year period among ART initiators and those not yet eligible for ART [15]. The cohort study was given ethics approval by the University of KwaZulu-Natal (ref BF083/08) and London School of Hygiene and Tropical Medicine (ref 5413).

### Outcomes

We calculate crude partnership acquisition and dissolution rates, and consider the impact of ART on the following five outcomes:

(1) Partner acquisition: Participants were considered at risk of acquiring a new partner from study enrolment and censored at last study visit.
(2) Partnership dissolution: All ongoing partnerships at enrolment and new partnerships were considered at risk of dissolution from the enrolment date and reported date of relationship start, respectively. Dissolution date was calculated as the date of last sex prior to break up if the participant reported sex with this partner since prior visit, or the date of the previous visit if this partnership was reported as ongoing at the previous visit and there had been no sex within this partnership between the previous visit and break-up.
(3) Frequency of sexual activity in the last month: The question ‘How many times have you had sexual intercourse with this partner in the last month?’ was asked for each partner.
(4) Sexual activity in the last month: The frequency of sexual activity in the last month response was coded into a binary indicator representing sex in the last month: yes (1) versus no (0).
(5) Unprotected sex in the last month: Those who were sexually active in the last month, were asked ‘On how many of these occasions did you and your partner use condoms throughout?’. A binary indicator represented unprotected sex in last month (1) versus condoms were used in all reported sex acts (0).

### Statistical analyses

R version 3.1.3 was used for all analyses [22]. Partnership acquisition and dissolution rates were calculated using the R package ‘epiDisplay’ [23]. The ‘survival’ and ‘coxme’ R packages [24–26] were used to fit multivariable Cox regression models [27,28] with and without frailties, and to test the proportional hazards assumption. For the acquisition model, we used a counting process formulation extension of the Cox model by Andersen and Gill [29] to incorporate all acquisitions observed during follow-up including repeated events within an individual. The time at risk for each individual is calculated as time since enrolment or last event, breaking the total time at risk for any individual with multiple events into multiple intervals of risk. For dissolution, one record per partnership was used, allowing partnership level covariates to vary between partnerships for a participant. Initially, Cox models with frailties were fitted to allow individual random effects. However, the variance of frailties was not statistically significant and a robust variance (WLW estimator, [30]) was used instead to account for clustering within individuals.

Generalized linear mixed models with a logit link, using the ‘lme4’ R package [31,32], were used to model the odds of sexual activity in the last month and the odds of unprotected sex among partnerships sexually active in the last month, with individual random effects to capture variation between participants. Finally, in order to model the number of sex acts in the last month in ongoing partnerships (count data), we fitted a negative binomial model with random effects and a log link function (log-linear model) using the ‘glmmADMB’ R package [33,34]. The negative binomial model was used to account for over-dispersion and the relatively high number of zeros in the outcome. Initially included as dummy variables representing each visit, estimates suggested that time could be reasonably represented by one indicator in each model: 6 or more months after first report versus first report of partnership.

Individual and partnership characteristics, and whether a relationship was ongoing at enrolment, were considered in models for the outcome of dissolution and all sexual activity outcomes, that is, all partnership-level analyses. For acquisition, only individual-level variables were considered.

In building a multivariable model for each outcome, we used a combination of forward and backward selection, and both *P* value and akaike information criterion criteria to identify significant predictors in a final parsimonious model. In all final models, we controlled for age (four categories: 18–21 years, 22–29 years, 30–40 years and >40 years), time in the study and participant's sex. We had previously shown that, controlling for sex, there were few differences at baseline between the two ART groups. However, the pre-ART group was significantly more likely to have been sexually active in the last month than the ART-eligible group, suggesting they might be more physically well [16]. We considered an interaction term between ART group and time in the study to allow the association between ART and each sexual behaviour outcome to vary over time [35]. Given that these analyses were at the partnership level, first report of partnership was synonymous with enrolment for most but not all of the partnerships. The analyses for each sexual behaviour outcome were repeated among partnerships ongoing at enrolment only and the results remained virtually unchanged. Thus, we refer to the time of first report of partnership as enrolment and time after first report as ‘during follow-up.’

A small number in the ART-eligible group never started ART while in follow-up. Similarly, a small proportion of the pre-ART group started ART during the study. Analyses for each outcome were repeated excluding those in the ART-eligible group who never started ART and censoring those in the pre-ART group who became ART-eligible at their ART initiation date. Exclusion of this subset did not substantively change the results; therefore, we kept the larger sample size for all analyses to increase power.

Descriptive analyses examined disclosure and knowledge of partner status across ART groups at enrolment and over time, among ongoing partnerships and new partnerships separately.

## Results

Six hundred and thirty-two participants were enrolled, 385 in the ART-eligible group (37% men) and 247 in the pre-ART group (14% men). The CD4^+^ test result used to define enrolment group was a median 22 days before enrolment, inter-quartile range (IQR 15, 36) for the ART-eligible group and 16 days (IQR 14, 27) for the pre-ART group, and all were ART-naive prior to that CD4^+^ test. Median CD4^+^ cell counts at enrolment were 133 cells/μl (IQR 76, 175) and 632 cells/μl (IQR 559, 768) for the ART-eligible and pre-ART groups, respectively. Median age and IQR were 35 years (29, 43) and 34 years (27, 43) in the ART-eligible and pre-ART groups, respectively. The median duration of follow-up was 2.97 years (IQR 2.44, 3.02) and 2.87 years (IQR 1.99, 3.01, *P* = 0.007), and the median lifetime number of sexual partners was 3 (IQR 2, 6) and 3 (IQR 2, 4) for ART-eligible and pre-ART groups, respectively.

At enrollment, 487 ongoing partnerships were reported among 467 participants (270 among ART-eligible and 197 pre-ART). Fourteen participants were in more than one partnership at enrolment (11 ART-eligible and 3 pre-ART). Five hundred and eighty-seven participants had at least one follow-up visit and an opportunity to report a change in partnership status. During the study, 68 participants died (84% of these were in the ART-eligible group), 19 out-migrated from the health district and were lost to follow-up (32% of these were in the ART-eligible group), one (ART-eligible) went to prison and was unavailable for interview and 33 (64% of these were in the ART-eligible group) refused to continue follow-up before their final visit. Ninety-four percent of the ART-eligible group started ART, a median 15 days after enrolment (IQR 7, 28), and 39 (16%) of the pre-ART group progressed to become ART-eligible and initiated ART during the analysis period, a median 19 months after enrolment (IQR 13, 24).

### Partner acquisition

In all, 161 new partnerships were observed among 132 individuals during follow-up. In the final multivariable Cox model (Table 1), participants had an increased hazard of acquiring a new partner if they were below 30 years old, with those aged 18–21 years almost two times more likely compared to 22–29-year-olds; had more than three lifetime partners, had no partner at enrolment, had ever taken alcohol, and had not disclosed their HIV status to anyone. There was no significant difference in acquisition hazard by ART group or sex.

**Table 1:** Acquisition rates, and univariable and multivariable Cox regression model results for time to partnership acquisition (*N* = 587).

Variable	Events^a^^,^^b^ (*N* = 161)	Person-years at risk	Rate per 100 person-years) (95% CI)	Unadj. HR^c^	CI	Adjusted HR^d^	CI	Wald *P* value
ART group								
Pre-ART	62	597.51	10.38 (7.96, 13.30)	1.00		1.00		
ART-eligible	99	947.15	10.45 (8.50, 12.73)	1.07	(0.76, 1.49)	0.97	(0.70, 1.35)	0.87
Sex								
Female	120	1138.67	10.54 (8.74, 12.60)	1.00		1.00		
Male	41	405.98	10.10 (7.25, 13.70)	0.96	(0.64, 1.42)	0.62	(0.41, 0.94)	0.025
Age (years)								
18–21	18	64.15	28.06 (16.63, 44.35)	1.89	(1.18, 3.02)	1.96	(1.25, 3.06)	<0.001
22–29	61	387.46	15.74 (12.04, 20.22)	1.00		1.00		
30–39	60	573.60	10.46 (7.98, 13.46)	0.73	(0.51, 1.04)	0.71	(0.50, 1.01)	
40+	22	519.45	4.24 (2.65, 6.41)	0.28	(0.16, 0.50)	0.21	(0.12, 0.36)	
Partner status at enrolment								
Ongoing partner^e^	100	1156.55	8.65 (7.04, 10.52)	1.00		1.00		
No partner	61	388.11	15.72 (12.02, 20.19)	1.81	(1.29, 2.52)	2.50	(1.84, 3.40)	<0.001
Has previously disclosed HIV status to anyone								
No	25	185.13	13.50 (8.74, 19.93)	1.00		1.00		
Yes	136	1359.53	10.00 (8.39, 11.83)	0.74	(0.45, 1.20)	0.58	(0.37, 0.89)	0.014
Ever used alcohol								
No	63	820.91	7.67 (5.90, 9.82)					
Yes	98	723.74	13.54 (10.99, 16.50)	1.76	(1.27, 2.45)	1.70	(1.18, 2.44)	0.004
No. of lifetime partners^f^								
≤3	75	907.40	8.27 (6.50, 10.36)	1.00		1.00		
>3	81	600.64	13.49 (10.71, 16.76)	1.63	(1.18, 2.26)	1.81	(1.27, 2.58)	0.001

ART, antiretroviral therapy; CI, confidence interval; HR, hazard ratio.

^a^In a few instances, participants reported getting back together during follow-up with the person they had reported as their most recent but not ongoing partnership at enrolment. A few other participants reported a new partner but they never became sexually active with them. These were not considered new acquisitions in the analysis.

^b^Twenty-four partnerships were concurrent, that is, reported to have started while the participant was in at least one other ongoing partnership.

^c^No other variables were significant in univariable models.

^d^The final model did not violate the proportional hazards assumption, global test *P* = 0.09. Fitting a model with Gaussian individual frailties, the estimated variance was not found significantly different from zero (*P* value = 0.92), suggesting very little variation between individuals.

^e^There were two groups of participants with ongoing partners at baseline, those who were recently sexually active and those who were abstaining from sex with their partners for various reasons. The number of new acquisitions, acquisition rates and 95% CIs for these two groups were: 90/1054.3 = 8.54 (6.86, 10.5) and 10/102.25 = 9.78 (4.69, 17.99) respectively and were not statistically different from each other.

^f^Missing values for 15 participants.

### Partnership dissolution

In all, 565 partnerships (404 ongoing at enrolment and 161 new during follow-up) among 466 participants contributed to this analysis. One hundred and ninety-two partnerships dissolved during follow-up. In the final multivariable Cox model (Table 2), partnerships had an increased hazard of dissolution if partners were not residing together, if the quality of the relationship was low and if the participant had ever taken alcohol. Partnerships of more than 5 years duration at first report were significantly less likely to dissolve than partnerships of less than 1 year duration. There was no significant difference in dissolution hazard between ART groups, men and women, or by age.

**Table 2:** Dissolution rates, and univariable and multivariable Cox regression model results for time to partnership dissolution (*N* = 565 partnerships, 466 participants).

Variable	Events^a^	Partnership-years at risk	Rate per 100 partnership-years) (95% CI)	Unadjusted HR^b^	CI	Adjusted HR^c^	CI	Wald p-value
ART group								
Pre-ART	76	416.88	18.23 (14.36, 22.82)	1.00		1.00		
ART-eligible	116	642.20	18.06 (14.93, 21.66)	1.01	(0.75, 1.34)	0.97	(0.73, 1.29)	0.840
Sex								
Female	134	736.00	18.21 (15.25, 21.56)	1.00		1.00		
Male	58	323.08	17.95 (13.63, 23.21)	0.99	(0.73, 1.37)	1.07	(0.73, 1.56)	0.745
Age (years)								
18–21	12	42.49	28.25 (14.60, 49.34)	1.05	(0.61, 1.81)	0.86	(0.46, 1.61)	0.640
22–29	73	284.79	25.63 (20.09, 32.23)	1.00		1.00		
30–39	69	425.82	16.20 (12.61,20.51)	0.65	(0.47, 0.90)	0.82	(0.59, 1.14)	
40+	38	305.99	12.42 (8.79, 17.05)	0.51	(0.34, 0.75)	0.77	(0.49, 1.23)	
Ever used alcohol								
No	78	535.60	14.56 (11.51, 18.18)	1.00		1.00		
Yes	114	523.48	21.78 (17.96, 26.16)	1.46	(1.10, 1.94)	1.38	(1.02, 1.88)	0.040
Partner lives								
With participant	53	524.09	10.11 (7.58, 13.23)	1.0		1.0		
Not with participant	139	530.77	26.19 (22.02, 30.92)	2.48	(1.81, 3.40)	1.84	(1.26, 2.68)	0.001
Partnership duration^d^								
Less than 1 year	88	284.64	30.92 (24.80, 38.09)	1.00		1.00		
1–5 years	58	297.39	19.50 (14.81, 25.21)	0.67	(0.48, 0.95)	0.77	(0.54, 1.11)	0.001
More than 5 years	46	477.05	9.64 (7.06, 12.86)	0.34	(0.24, 0.49)	0.47	(0.31, 0.71)	
Relationship quality^e^								
Lowest quartile	71	275.37	25.78 (20.14, 32.52)	1.00		1.00		
Second quartile	68	425.35	15.99 (12.41, 20.27)	0.63	(0.45, 0.88)	0.70	(0.50, 0.99)	
Third quartile	39	281.46	13.86 (9.85, 18.94)	0.54	(0.37, 0.80)	0.67	(0.44, 1.02)	
Fourth quartile	8	65.85	12.15 (5.24, 23.94)	0.47	(0.22, 0.98)	0.46	(0.22, 0.96)	0.061

ART, antiretroviral therapy; CI, confidence interval; HR, hazard ratio.

^a^Seventy-four participants had more than one relationship at risk of dissolution during the study: 62 with two partnerships, 9 with three partnerships, 2 with four and one with five partnerships. Of the 192 partnerships that dissolved, 133 were ongoing at enrolment among 129 participants, and 59 were new partnerships among 51 participants.

^b^Other variables measured at first report of partnership that were significant in univariable models: having tested HIV-positive less than 1 year before enrolment, higher perceived stigma, little reliance on family and friends, and reporting that a condom was used at first sex within the partnership were all associated with a greater hazard of dissolution. Knowing someone on antiretroviral drugs, complete knowledge about antiretroviral drugs, knowing their partner's HIV status, and having disclosed their own HIV status to their partner was associated with a lower hazard of dissolution. Partnerships that started after enrolment had a significantly higher hazard of dissolution.

^c^The final model did not violate the proportional hazards assumption, global test *P* = 0.48. The estimated variance of individual frailties was not found significantly different from zero (*P* value = 0.91), suggesting no significant variation between participants.

^d^Relationship duration represents how long the partnership had been ongoing at the time of first report of the partnership in the study, and is not time-varying.

^e^The highest quartile represents the 25% of partnerships with the greatest reported social support from their partner, a proxy for higher relationship quality.

### Sexual activity in the last month

In the final multivariable model (Table 3), the estimated odds of having had sex in the last month for participants in the ART-eligible group were approximately half of those in the pre-ART group at enrolment [adjusted odds ratio (aOR) 0.51, 95% confidence interval (CI) 0.31, 0.81]. In contrast, during follow-up, the OR was 0.91 (95% CI 0.61, 1.34). The model also estimated a lower odds for participants reporting that they had not used condoms (during last sex with partner or never used), participants believing that their partner had sex with someone else and participants not living with their partner. On the contrary, knowing partner's HIV status and wanting to have more children were associated with higher odds of having sex in the last month. Individuals in a new relationship rather than a relationship ongoing at enrolment and those who had argued with their partner recently were also more likely to be sexually active in the last month.

**Table 3:** Univariable and multivariable logistic regression models of the odds of sexual activity in the last month among partnerships that were ongoing in the month before interview (*N* = 640 partnerships, 515 participants, 2363 observations^a^).

Variable	*N* (% reporting outcome)	Unadjusted OR^b^	95% CI	Adjusted OR^c^	95% CI	Wald *P* value
ART group						
Pre-ART	956 (82)	1.00		1.00		
ART-eligible	1407 (80)	0.80	(0.58, 1.09)	0.51	(0.31, 0.81)	0.005
Time (months)						
First report of partnership	613 (76)	1.00		1.00		
≥6 months after first report	1750 (85)	3.13	(2.47, 3.97)	1.35	(0.88, 2.07)	0.17
Interaction: ART group × time^d^				1.79	(1.05, 3.05)	0.031
Sex						
Female	1646 (80)	1.00		1.00		
Male	717 (83)	1.23	(0.89, 1.71)	0.70	(0.47, 1.03)	0.071
Age (years)						
18–21	98 (67)	0.51	(0.26, 1.02)	0.52	(0.25, 1.10)	0.094
22–29	651 (77)	1.00		1.00		
30–39	972 (82)	1.43	(1.00, 2.04)	1.28	(0.86, 1.89)	
40+	642 (83)	1.38	(0.93, 2.04)	1.33	(0.84, 2.11)	
Condom used at last sex						
Yes	1923 (84)	1.00		1.00		<0.001
No, but has used condoms with partner	209 (75)	0.49	(0.33, 0.73)	0.57	(0.37, 0.88)	
No, never used condoms with partner	231 (56)	0.15	(0.11, 0.21)	0.23	(0.15, 0.34)	
Partner had sex with others in past 6 months						
No	1329 (86)	1.00		1.00		<0.001
Yes/I think so	1034 (74)	0.42	(0.33, 0.53)	0.57	(0.43, 0.75)	
Partner lives						
With participant	1269 (87)	1.00		1.00		<0.001
Not with participant	1094 (73)	0.35	(0.27, 0.46)	0.39	(0.28, 0.54)	
Recently argued						
No	1864 (80)	1.00		1.00		0.009
Yes	499 (82)	1.13	(0.84, 1.51)	1.56	(1.12, 2.17)	
Want more children						
No	1603 (80)	1.00		1.00		0.001
Yes	760 (82)	1.34	(1.02, 1.77)	1.67	(1.23, 2.27)	
Know partner's HIV status						
No	824 (72)	1.00		1.00		0.077
Yes	1539 (85)	2.52	(1.94, 3.28)	1.33	(0.97, 1.81)	
Partnership type						
Ongoing at enrolment	1950 (81)	1.00		1.00		0.001
New	413 (82)	1.63	(1.14, 2.35)	1.98	(1.33, 2.96)	

ART, antiretroviral therapy; CI, confidence interval; OR, odds ratio.

^a^Seventy-five partnerships contributing to this analysis were ongoing at first report, but had no further follow-up of the participant or no further report of that partnership and therefore could not contribute to the time to dissolution analysis (Table 2).

^b^In univariable analysis, higher odds of having sex in last month was also associated with being employed, having self-initiated testing for a reason other than being sick, having disclosed HIV status to partner and higher relationship quality. In contrast, spending little or no time with friends, and the involvement of alcohol in last sex were associated with lower odds of having sex in the last month.

^c^Also adjusted for clinic where recruitment for the study occurred. Estimated random-effects variance = 1.061 (24% of total variance).

^d^The estimated odds ratio of sexual activity for the ART-eligible group after first report compared to the pre-ART group after first report is aOR 0.91 (0.61, 1.34), calculated by exponentiating the sum of the ART group main effect and interaction effect coefficients.

### Unprotected sex acts in the last month

Table 4 presents the final model for the odds of unprotected sex in the last month among partnerships that reported sexual activity in the last month. There were no significant differences between the two ART groups at enrolment (aOR 1.10, 95% CI 0.57, 2.12), whereas during follow-up, the odds of unprotected sex were significantly lower for partnerships of ART-eligible participants compared to pre-ART participants (aOR 0.26, 95% CI 0.15, 0.43). Significantly lower odds of unprotected sex in the last month were also associated with the participant having more equitable gender norms, not living with their partner and having disclosed their HIV status to the partner. Higher odds of unprotected sex were associated with the involvement of alcohol at last sex, ever having had unwanted sex within the partnership, desire to have (more) children with partner and the partner having ever performed a physical act of violence towards the participant. Unprotected sex with a partner who is HIV-negative or has unknown HIV status is considered risky sex. Of the total sexual acts by participants in the ART-eligible group, 5.5% were categorized as risky acts, compared to 13.2% of the sexual acts in the pre-ART group (*P* < 0.0001). Thirty-three percentage (186) of the 565 partnerships reported unprotected sex at least once, 104 (56%) of them had risky sex, the rest had unprotected sex with a partner known to have HIV.

**Table 4:** Logistic regression models of the odds of unprotected sex in the last month among partnerships that reported sexual activity in the month before interview (*N* = 551 partnerships, 457 participants, 1902 observations).

Variable	*N* (% reporting outcome)	Unadjusted OR^a^	(95% CI)	Adjusted OR^b^	95% CI	*P*
ART group						
Pre-ART	782 (21)	1.00		1.00		
ART-eligible	1120 (11)	0.35	(0.23, 0.53)	1.10	(0.57, 2.12)	0.77
Time (months)						
First report of partnership	408 (23)	1.00		1.00		
≥6 months after first report	1494 (13)	0.44	(0.32, 0.62)	1.11	(0.67, 1.85)	0.69
Interaction: ART group × time^d^				0.23	(0.11, 0.47)	<0.001
Sex						
Female	1315 (17)	1.00		1.00		
Male	587 (11)	0.50	(0.30, 0.83)	0.82	(0.47, 1.41)	0.47
Age (years)						
18–21	66 (29)	2.63	(0.91, 7.57)	2.38	(0.82, 6.93)	0.09
22–29	505 (17)	1.00		1.00		
30–39	799 (16)	0.98	(0.59, 1.66)	0.95	(0.55, 1.65)	
40+	532 (11)	0.66	(0.37, 1.21)	0.59	(0.31, 1.13)	
Alcohol involved in last sex^c^						
No	1798 (14)	1.00		1.00		<0.001
Yes	104 (34)	4.32	(2.46, 7.60)	3.29	(1.79, 6.05)	
Gender norms^e^						
Lowest quartile	661 (18)	1.00		1.00		<0.001
Second quartile	449 (20)	1.23	(0.84, 1.80)	1.17	(0.78, 1.75)	
Third quartile	395 (10)	0.42	(0.26, 0.68)	0.44	(0.27, 0.72)	
Fourth quartile	397 (9)	0.36	(0.21, 0.63)	0.38	(0.22, 0.66)	
Disclosed HIV status to partner						
No	250 (27)	1.00		1.00		0.001
Yes	1652 (13)	0.32	(0.20, 0.50)	0.43	(0.25, 0.72)	
Partner lives						
With participant	1105 (16)	1.00		1.00		0.009
Not with participant	797 (14)	0.77	(0.52, 1.14)	0.56	(0.37, 0.87)	
Partner insists to have sex when participant does not want to						
No	1472 (13)	1.00		1.00		0.071
Yes	430 (21)	1.63	(1.12, 2.37)	1.44	(0.97, 2.13)	
Want more children						
No	1281 (13)	1.00		1.00		0.001
Yes	621 (20)	1.98	(1.38, 2.84)	1.92	(1.31, 2.82)	
Partner performed physical act of violence to participant						
No	1806 (15)	1.00		1.00		0.063
Yes	96 (24)	2.69	(1.42, 5.08)	1.94	(0.96, 3.91)	

ART, antiretroviral therapy; CI, confidence interval; OR, odds ratio.

^a^In univariable analysis, higher odds of unprotected sex in the last month was also associated with higher levels of perceived stigma and spending little or no time with friends. In contrast, being a male participant, having disclosed HIV status to at least one person and knowing partner's HIV status were associated with lower odds of unprotected sex.

^b^Estimated random effects variance = 2.014 (38% of total variance).

^c^This partnership-level variable was time-varying and who had taken alcohol varied (male or female partner or both), but numbers were too small to explore according to who had taken it separately. On average, 75% of the reports of alcohol at last sex across visits were that the partner had taken alcohol.

^d^The estimated odds ratio of unprotected sex in the last month for the ART-eligible group after first report compared to the pre-ART group after first report is aOR 0.26 (0.15, 0.43), calculated by exponentiating the sum of the ART group main effect and interaction effect coefficients.

^e^The highest quartile represents the 25% of the cohort with the most equitable gender norms.

### Frequency of sex acts in the last month

In the final multivariable negative binomial model (Table 5), the number of sex acts was 23% lower among the ART-eligible group compared to the pre-ART group at enrolment [adjusted incidence rate ratio (aIRR) 0.77, 95% CI 0.65, 0.91], whereas during follow-up, the ratio was no longer significantly different from 1.0 (aIRR 0.97, 95% CI 0.88, 1.08). The number of sex acts in the last month was higher when more children were wanted and among new partnerships. The number of sex acts in the last month was lower with more equitable gender norms, when condoms were not used at every sex act, when unwanted sex had ever happened within the partnership, when the participant believed their partner had sex with others and when the couple were not living together.

**Table 5:** Univariable and multivariable negative binomial models of the number of sex acts in the last month among partnerships that were ongoing in the month before interview (*N* = 640 partnerships, 515 participants, 2370 observations)^a^.

Variable	Unadjusted IRR^b^	(95% CI)	Adjusted IRR^c^	95% CI	*P*
ART group					
Pre-ART	1.00		1.00		
ART-eligible	0.93	(0.83, 1.03)	0.77	(0.65, 0.91)	0.002
Time (months)					
First report of partnership	1.00		1.00		
≥6 months after first report	1.54	(1.41, 1.68)	1.22	(1.07, 1.38)	0.002
Interaction: ART group × time^d^			1.26	(1.07, 1.50)	0.007
Sex					
Female	1.00		1.00		
Male	1.14	(1.02, 1.27)	1.02	(0.92, 1.14)	0.71
Age (years)					
18–21	0.85	(0.66, 1.11)	0.89	(0.78, 1.13)	0.220
22–29	1.00		1.00		
30–39	1.09	(0.96, 1.23)	1.00	(0.89, 1.12)	
40+	1.02	(0.89, 1.17)	0.89	(0.78, 1.01)	
Gender norms^e^					
Lowest quartile	1.00		1.00		<0.001
Second quartile	0.78	(0.71, 0.85)	0.85	(0.78, 0.93)	
Third quartile	0.72	(0.65, 0.80)	0.76	(0.69, 0.84)	
Fourth quartile	0.74	(0.67, 0.82)	0.80	(0.72, 0.90)	
Frequency of condom use with this partner in past 6 months					
Always	1.00		1.00		<0.001
Never/sometimes	0.74	(0.69, 0.80)	0.86	(0.79, 0.93)	
Partner insists to have sex when participant does not want to					
No	1.00		1.00		0.044
Yes	0.86	(0.78, 0.94)	0.91	(0.83, 1.00)	
Partner had sex with others in past 6 months					
No	1.00		1.00		<0.001
Yes/I think so	0.75	(0.70, 0.81)	0.86	(0.80, 0.93)	
Partner lives					
With participant	1.00		1.00		<0.001
Not with participant	0.72	(0.66,0.78)	0.75	(0.68, 0.82)	
Want more children					
No	1.00		1.00		0.003
Yes	1.11	(1.02, 1.20)	1.13	(1.04, 1.23)	
Partnership type					
Ongoing at enrolment	1.00		1.00		<0.001
New	1.18	(1.05, 1.32)	1.24	(1.11, 1.39)	

ART, antiretroviral therapy; CI, confidence interval; IRR, incidence rate ratio.

^a^The number of observations for each variable is reported in Table 3 as the same partnership observations contribute to both analyses.

^b^Variables found significant in univariable analysis only: higher incidence rate ratio (IRR) associated with being male, knowing anyone on antiretroviral drugs before enrolment, higher levels of perceived stigma, complete antiretroviral drug knowledge, spending little or no time with family, knowledge of partner's HIV status, having disclosed HIV status to partner, more than three lifetime partners and higher relationship quality scores. Lower IRR was associated with greater reliance on family/friends when having a serious problem and the participant had ever performed a physical act of violence to their partner.

^c^Also adjusted for clinic where recruitment for the study occurred. Estimated random-effects variance = 0.098.

^d^The estimated incidence rate ratio of the number of sex acts in the last month for the ART-eligible group after first report compared to the pre-ART group after first report is aIRR 0.97, 95% CI (0.88, 1.08); calculated by exponentiating the sum of the ART group main effect and interaction effect coefficients.

^e^The highest quartile represents the 25% of the cohort with the most equitable gender norms.

### Disclosure to partner and knowledge of partner status

Among ongoing partnerships at enrolment, 346 (71%) participants had already disclosed their HIV status to their partner and 63 more (13%) disclosed during follow-up, with no difference between ART groups (*P* = 0.79 and *P* = 0.22, respectively). Two hundred and twenty participants (45%) knew their partner status at enrolment (*P* = 0.11 for this proportion across the ART groups), and a further 114 (23%) learned their partner's status during follow-up – 80 (28%) in the ART-eligible group and 34 (17%) in the pre-ART group (*P* = 0.006). Among partnerships initiated during follow-up, 93 (58%) had disclosed their HIV status to their partner and 61 (38%) knew their partner's HIV status by the time of first report of the new relationship, and there was no difference by ART group (*P* = 0.16 and *P* = 0.32, respectively). A further 20 (12%) disclosed their HIV status after first report while still in relationship and study follow-up, and 18 (11%) learned their partner's HIV status.

## Discussion

In this long-term follow-up study, being on ART was not associated with increased partner acquisition or partnership dissolution rates. Partner acquisition rate estimates in this study are two or three times lower than those estimated for the general population in the same area; in contrast, dissolution rate estimates were three or four times higher [36]. It is difficult to compare the proportion of partnerships that dissolved in this study with other studies because of differences in the study population and duration of follow-up. Both a study in Nairobi with 1–2 years follow-up and a randomized trial measuring life events 6 months after voluntary counselling and testing in Kenya, Tanzania and Trinidad reported approximately one quarter of partnerships had dissolved, compared to 34% of all couples in our study [4,6]. These previous studies did not report dissolution rates.

By ART group, we observed no difference in HIV disclosure and knowledge of partner HIV status. The proportion disclosing to, or knowing the HIV status of a partner, did not differ between new and established partnerships either, contrary to our a priori hypothesis that HIV disclosure may be easier in new partnerships after linkage to care. Being on ART was not associated with increased sexual risk behaviours, consistent with other shorter-term studies in Africa [8,37–39], which have also shown reductions in sexual risk behaviours with ART. Indeed, reports of unprotected sex were significantly lower among the ART group during follow-up, a reassuring finding given the WHO recommends ART initiation upon HIV diagnosis [40], which will result in many more people on ART.

The study provides evidence that relationship-level characteristics determine partnership dissolution, the odds and frequency of sexual activity and the odds of unprotected sex, all of which influence onward HIV transmission. Living with a partner, longer partnership duration and higher reported relationship quality were associated with lower risk of partnership dissolution. Living with a partner and wanting more children with that partner were associated with increased odds and frequency of sexual activity, and higher odds of unprotected sex. Other relationship-level characteristics were found to be significantly associated with at least one of the sexual behaviour outcomes, including believing their partner had not had sex with others in the past 6 months (higher odds and frequency of sexual activity) and being in a new partnership (higher odds and frequency of sexual activity).

The odds of unprotected sex were higher within partnerships if a partner had ever insisted to have sex when the participant did not want to, had ever been physically violent towards the participant, alcohol was involved in last sex and the participant had inequitable gender norms. The odds of unprotected sex were lower when the participant had disclosed their HIV status to their partner. Kerridge *et al*[41] reported similar findings regarding alcohol use and unprotected sex in Uganda, and suggested that HIV programmes promoting condom use combine alcohol reduction messaging and address gender norms. Clinic staff could tailor messages regarding HIV disclosure, condom use and partner testing by routinely asking questions about ongoing and new partners. Staff could also identify individuals needing support around physical and sexual violence. Discussing relationships in the clinic setting could also identify individuals who wants children with their partner and would benefit from fertility support services.

Advancing our knowledge of partnerships and sexual behaviour in an HIV care context, this study has some limitations. All interviews were face-to-face or by phone, which may have resulted in social desirability bias, particularly in the reporting of condom use and HIV disclosure [42–44]. In addition, this study population may not be representative with respect to partnership acquisition and dissolution of individuals with HIV who are not engaged in the HIV clinic. Capturing sexual activity, frequency of sex acts and condom coverage over the short period of 1 month before interview limited several analyses to focus only on those partnerships that were ongoing in the month before interview and these may not have been representative of all partnerships that occurred in the study. However, asking participants about sexual acts more than 1 month ago would have potentially introduced recall bias. Neither disease stage at enrolment nor history of illness in the year prior to enrolment were available; thus we are unable to explore to what extent the effect of ART was due to an overall health improvement in the ART group.

In Africa, there is much more that can be provided by way of support for HIV disclosure and sexual risk reduction, and promotion of couples-testing and positive couple relationships over the course of HIV care. The repeated interaction with the clinics required for HIV treatment and CD4^+^ cell count and viral load measurement provide an opportunity for strategies similar to the ‘Making every contact count’ [45] advocated by National Health Service England which promotes delivery of brief advice at every opportunity to improve health and well being. With HIV now a chronic disease, this study highlights the opportunities to respond to changing partnership dynamics of individuals in HIV care services.

## Acknowledgements

We thank the individuals who participated in the study and the study team (Nompilo Myeni, Thabile Hlabisa, Nompilo Buthelezi, T.T. Khumalo, Khetiwhe Ngobese, Witness Ndlovu and Patrick Gabela) who made this work possible.

Funding: Data collection and both authors were supported by a Wellcome Trust fellowship (Grant # WT083495MA).

### Conflicts of interest

There are no conflicts of interest.
